# TCM-Blast for traditional Chinese medicine genome alignment with integrated resources

**DOI:** 10.1186/s12870-021-03096-1

**Published:** 2021-07-17

**Authors:** Zhao Chen, Jing Li, Ning Hou, Yanling Zhang, Yanjiang Qiao

**Affiliations:** 1 School of Chinese Materia Medica, Beijing University of Chinese Medicine, Yangguang South Avenue, Fangshan District, Beijing, 102488 China; 2 Research Center of TCM-Information Engineering, State Administration of Traditional Chinese Medicine of The Peoples Republic of China, Yangguang South Avenue, Fangshan District, Beijing, 102488 China

## Abstract

The traditional Chinese medicine (TCM) genome project aims to reveal the genetic information and regulatory network of herbal medicines, and to clarify their molecular mechanisms in the prevention and treatment of human diseases. Moreover, the TCM genome could provide the basis for the discovery of the functional genes of active ingredients in TCM, and for the breeding and improvement of TCM. The traditional Chinese Medicine Basic Local Alignment Search Tool (TCM-Blast) is a web interface for TCM protein and DNA sequence similarity searches. It contains approximately 40G of genome data on TCMs, including protein and DNA sequence for 36 TCMs with high medical value.The development of a publicly accessible TCM genome alignment database hosted on the TCM-Blast website (http://viroblast.pungentdb.org.cn/TCM-Blast/viroblast.php) has expanded to query multiple sequence databases to obtain TCM genome data, and provide user-friendly output for easy analysis and browsing of BLAST results. The genome sequencing of TCMs helps to elucidate the biosynthetic pathways of important secondary metabolites and provides an essential resource for gene discovery studies and molecular breeding. The TCMs genome provides a valuable resource for the investigation of novel bioactive compounds and drugs from these TCMs under the guidance of TCM clinical practice. Our database could be expanded to other TCMs after the determination of their genome data.

**Supplementary Information:**

The online version contains supplementary material available at 10.1186/s12870-021-03096-1.

## Background

Whole-genome sequencing of the plants that form the basis of traditional Chinese medicine (TCM) is an important means for gene discovery and cultivation, synthetic biology, drug discovery and molecular breeding involving TCMs [1–4]. The genomic sequence provides a valuable resource not only for fundamental and applied research, but also for evolutionary and comparative genomics analyses, particularly in TCMs [5–9].

Experimental and clinical studies have demonstrated that TCMs have a wide range of pharmacological properties such as anti-inflammatory, antiviral, antimicrobial, antioxidative, antifungal, antithrombotic, antihyperlipidemic, analgesic, antidiabetic, antidepressant, antiasthma and anticancer activities as well as immunomodulatory, antidiabetic, gastroprotective, hepatoprotective, neuroprotective and cardioprotective effects [10–18]. Genome sequencing and its annotations provide an essential resource for TCM improvement through molecular breeding [19–21] and for the discovery of useful genes for engineering bioactive compounds through synthetic biology approaches [1, 22–24]. The availability of these genomic resources will facilitate the discovery of medicinally and nutritionally important genes, the genetic improvement of TCMs [7, 21, 25] and the identification of novel drug candidates [26].

The Herbal Medicine Omics Database (http://herbalplant.ynau.edu.cn/html/Genomes/) has collected only 23 published genomes of medicinal herbs and there has been no continued update of the increased data since 2019. Only 14 kinds of medicinal plant genome data were provided in the Medicinal Plant Genomics Resource (http://medicinalplantgenomics.msu.edu). BLAST against plant genomes data (https://blast.ncbi.nlm.nih.gov/Blast.cgi?PROGRAM=blastn&_TYPE=BlastSearch&BLAST_SPEC=Plants_MV&LINK_LOC=blasttab&LAST_PAGE=blastp) included few types of medicinal plants, and the genome comparison of the most common edible plants was provided).

### Construction and content

Genome data of TCMs were originated from the Herbal Medicine Omics Database (http://herbalplant.ynau.edu.cn/html/Genomes/), the Medicinal Plant Genomics Resource (http://medicinalplantgenomics.msu.edu), and the BIG Data Center in Beijing Institute of Genomics, Chinese Academy of Sciences (http://bigd.big.ac.cn/gsa/statistics).

The genome data of Chinese medicinal materials originating from unlabeled references are from http://medicinalplantgenomics.msu.edu/, http://bigd.big.ac.cn/gsa/statistics.

The deployment strategy for TCM-Blast involves instantiating a provided Viroblast [27] that bundles the core components for TCM genome alignment. A user-friendly web interface to search the database has been implemented in PHP 7.0.32 (http://www.php.net) and deployed on an Apache 2.4.18 web server (http://www.apache.org/) and MySQL database server (https://www.mysql.com/) with Ubuntu 16.04 server (http://mirrors.aliyun.com/ubuntu-releases/16.04/). TCM-Blast had 36 TCMs genome datasets.

The information regarding TCM genome datasets is summarized in an online at the TCM-Blast website. The TCM genome data used in TCM-Blast were collected from the Herbal Medicine Omics Database (http://herbalplant.ynau.edu.cn/html/Genomes/), the Medicinal Plant Genomics Resource (http://medicinalplantgenomics.msu.edu), and the BIG Data Center in Beijing Institute of Genomics (http://bigd.big.ac.cn/gsa/statistics) (the further details on the genome data sources for the thirty-six TCMs, see Table 1). These data resources have been published in professional journals and plant gene databases by academic institutions or government departments merged with plant gene databases, with abundant data sources and reliable data quality. In addition to other data resources, this database in our study has the following advantages: 1) this database is currently the largest Chinese medicine genome database; 2) this database includes the plant genetic data of Chinese medicine sources; and 3) this database provides support for the TCM breeding, cultivation of TCMs and the discovery of active ingredients in TCMs.

**Table 1:** Data sources of thirty-six TCM genomes

*Latin name*	Pin Yin	Genome sequencing method	Reference
*Dendrobium Offcinale*	Tiepishihu	combining the second-generation Illumina Hiseq 2000 and third-generation PacBio sequencing technologies	Ref [8]
*Ginkgo Biloba*	Yinxing	Hiseq 2000/4000 platform	Ref [5]
*Erigeron Breviscapusd*	Dengzhanhua	Illumina sequencing and PacBio single-molecular real-time sequencing on the Illumina HiSeq platform	Ref [24]
*Panax Ginseng*	Sanqi	Illumina paired-end libraries for the whole-genome sequencing	Ref [26]
*Eucommia Ulmoides*	Duzhong	Illumina HiSeq, MiSeq short-read sequencing,and PacBio single molecular long-read sequencing	Ref [28]
*Punica Granatum*	Shiliu	Illumina paired-end reads of libraries	Ref [29]
*Dioscorea Routundata*	Shanyao	Illumina MiSeq platform, HiSeq 2500 platform	Ref [30]
*Ginseng*	Renshen	paired-end sequencing on the HiSeq X-Ten platform (Illumina)	Ref [21]
*Boea Hygrometrica*	Niuercao	whole-genome shotgun approach (Illumina HiSeq and Roche 454 platforms)	Ref [31]
*Jatropha Curcas*	Mafengshu	Illumina GAII and HiSeq	Ref [7]
*Glycyrrhiza Uralensis*	Gancao	short reads from Illumina and long reads from Pacific Biosciences sequencing	Ref [1]
*Moringa Oleifera*	Lamu	Illumina Hiseq2500TM	Ref [32]
*Salvia Miltiorrhiza*	Danshen	Illumina sequencing and PacBio sequencing,	Ref [33]
*Cannabis Sativa*	Dama	Illumina mate-pair library construction and sequencing	Ref [34]
*Mentha Longifoli*	Bohe	Illumina sequencing, Pacific Biosystems sequencing	Ref [22]
*Macleaya Cordata*	Boluohui	paired-end sequences on HiSeq 2000	Ref [35]
*Calotropis Gigantea*	Niuguajiao	Illumina HiSeq 2500	Ref [36]
*Rhodiola Rosea*	Hongjingtian	Illumina HiSeq 2000/4000 platform using a whole genome shotgun sequencing (WGS) strategy	Ref [37]
*Capsicum annuum*	Lajiao	Illumina HiSeq 2500	
*Lilium*	Baihe	Illumina HiSeq X Ten	
*Tupaia belangeri*	Baihuabaihe	Illumina HiSeq 2000	
*Arctium lappa*	Niubang	Illumina HiSeq X Ten	
*Anemone flaccida*	Ezhangcao	Illumina HiSeq 2000	
*Atropa belladonna*	Dianqie	RNA-seq for expression abundances	
*Digitalis purpurea*	Zihuayangdihuang	RNA-seq for expression abundances	
*Dioscorea villosa*	Changroumaoshuyu	RNA-seq for expression abundances	
*Echinacea purpurea*	Zizhuiyu	RNA-seq for expression abundances	
*Hoodia gordonii*	Hutieyaxianrenzhang	RNA-seq for expression abundances	
*Hypericum perforatum*	Guanyejinsitao	RNA-seq for expression abundances	
*Panax quinquefolius*	Xiyangshen	RNA-seq for expression abundances	
*Rauvolfia serpentina*	yinduluofumu	RNA-seq for expression abundances	
*Rosmarinus officinalis*	Midiexiang	RNA-seq for expression abundances	
*Valeriana officinalis*	Xiecao	RNA-seq for expression abundances	
*Camptotheca acuminata*	Huaxishu	Illumina sequencing platform	Ref [38]
*Catharanthus roseus*	Changchunhua	whole genome shotgun sequencing approach	Ref [39]
*Lepidium Meyenii*	Maca	Illumina HiSeq 2500 platform yielded 1.88 billion reads in ten paired-end libraries	Ref [40]

## Utility and discussion

### Overview of TCM-Blast

**Fig. 1:**
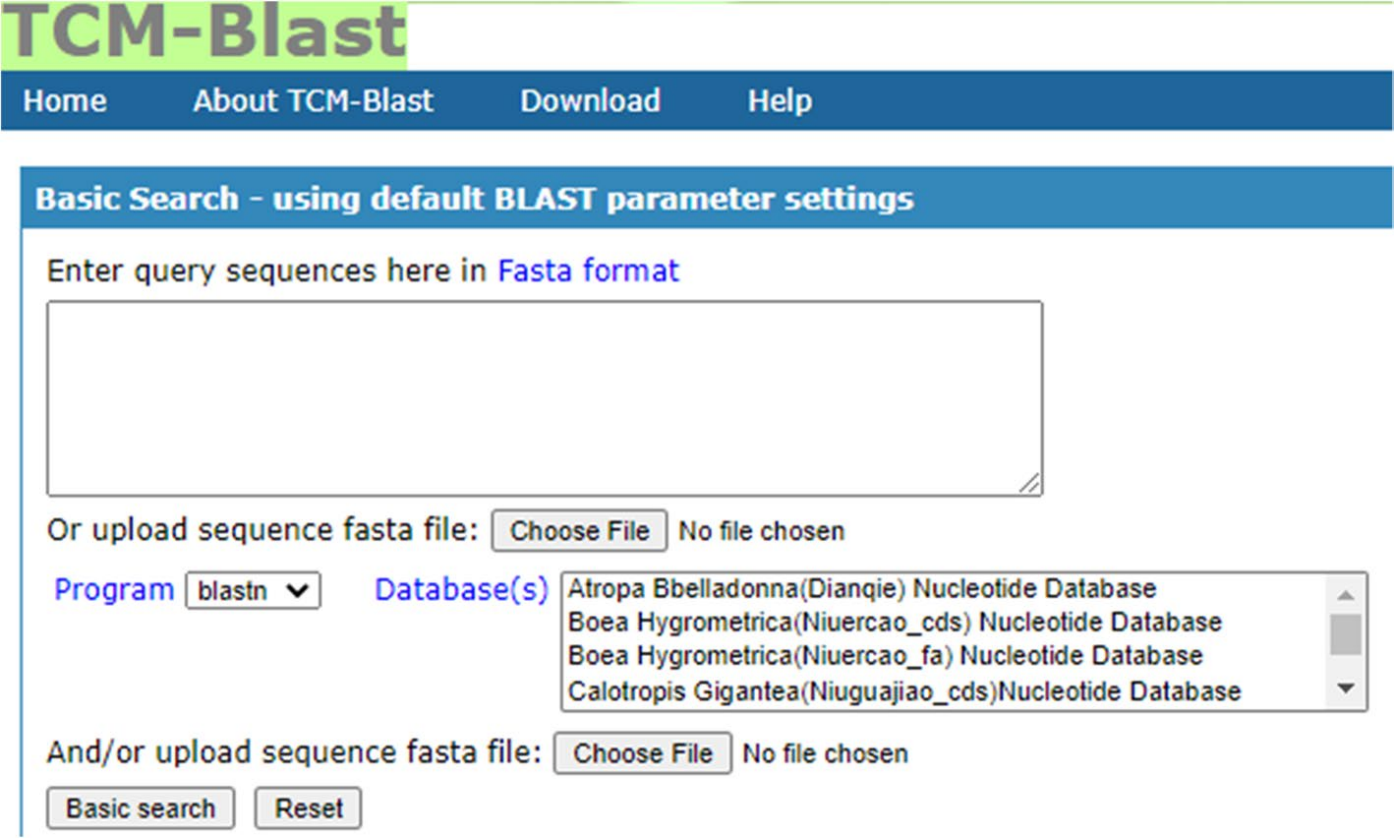
The homepage of TCM-Blast

We have developed TCM-Blast, a web-based database for TCM genome alignment (Fig. 1). TCM-Blast offers an interface to choose from TCM genome databases including TCM protein and DNA sequence datasets, which provide query functions with BLAST implementation [40]. TCM-Blast currently contains approximately 40 GB of TCM genome data, including the proteins and DNA sequences of 36 TCMs.

### The mains functions of TCM-Blast

The user can directly enter the query sequence directly by pasting into the query box or by uploading the sequence as a FASTA file from a local file. TCM-Blast provides multiple TCM sequence databases. Users can then select specific TCM genome databases to run different programs (blastn, blastp, blastx, tblastn, tblastx). TCM-Blast consists of five general BLAST form types [27, 41–43] for TCM genome data:

- blastn: search TCM nucleotide databases using a nucleotide query.
- blastp: search TCM protein databases using a protein query.
- blastx: search TCM protein databases using a translated nucleotide query
- tblastn: search TCM translated nucleotide databases using a protein query.
- tblastx: search TCM translated nucleotide databases using a translated nucleotide query

**Fig. 2:**
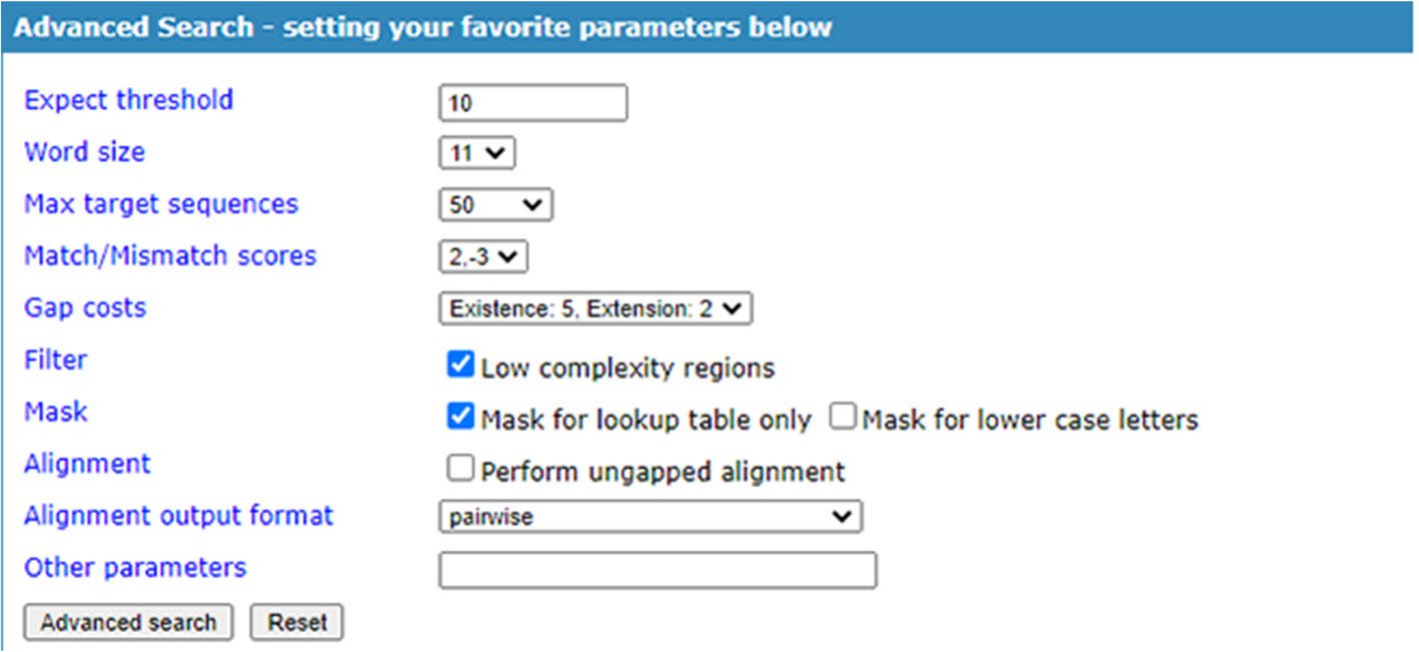
The setting for favorite parameters in TCM-Blast

**Fig. 3:**
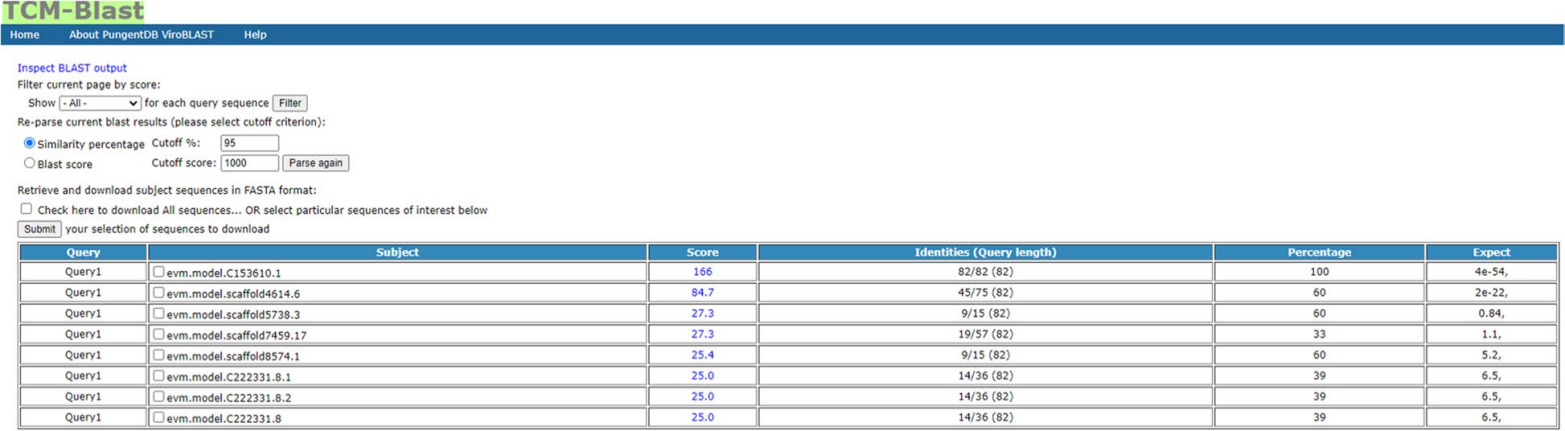
The BLAST result of TCM protein and DNA sequence similarity in TCM-Blast

TCM-Blast provides an optional search function for advanced users who need to collect more specific information (Fig. 2) with the ability to set different parameters, such as the expected threshold, word size, max target sequences, etc., to glean more specific information for users. The TCM-Blast sequence alignment results of the TCM genome sequence are displayed in the summary table, which contains the query sequence name, subject sequence name, subject source database, position score, identity percentage, and E value (Fig. 3).

### A case study of this database

**Fig. 4:**
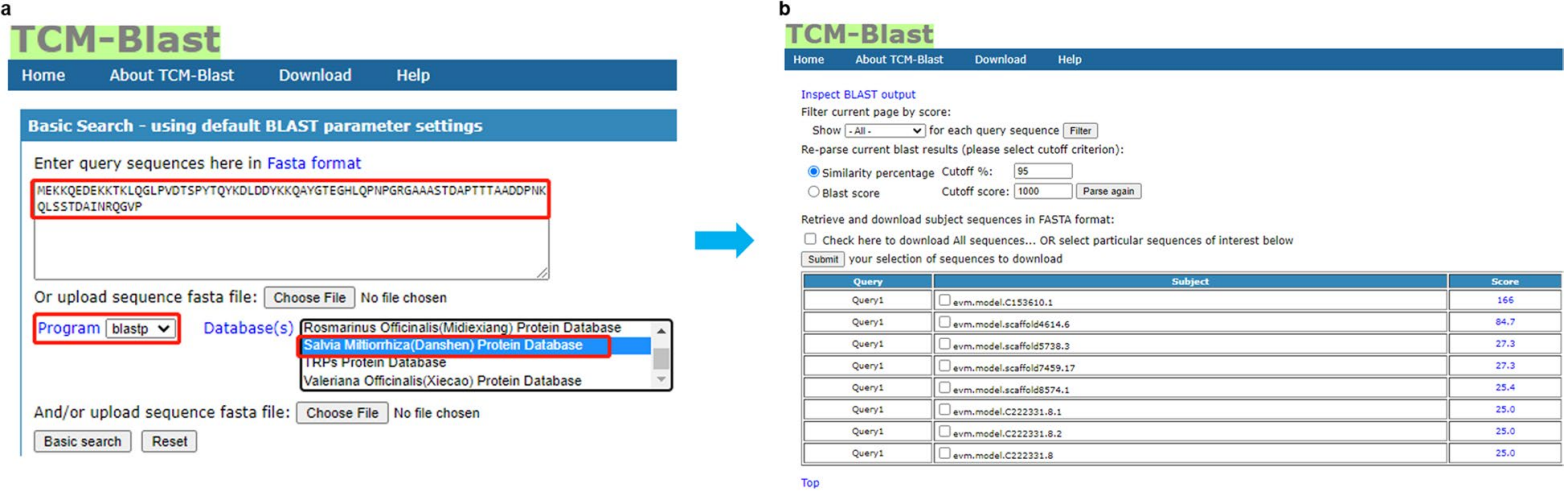
The BLAST result of *Salvia Miltiorrhiza* protein alignment with the input of *Salvia Miltiorrhiza* protein sequence fragment into TCM-Blast. In the first section (**a**), the user checks their protein sequence. In the second section (**b**), the BLAST results with the input protein sequence are briefly displayed in the table. Furthermore, detailed score information on this alignment can be checked by clicking each score item button

For example, the user can select the Salvia Miltiorrhiza protein database with the programs blastp and obtain their expected BLAST results by inputting the protein sequence. In Fig. 4, the user has input the protein sequence fragment:

“*MEKKQEDEKKTKLQGLPVDTSPYTQYKDLDDYKKQAYGTEGHLQPNPGRGAAASTDAPTTTAADDPNKQLSSTDAINRQGVP*” in the “Enter query sequences” box; selected the Salvia Miltiorrhiza protein database; and obtained the BLAST result by clicking the “Basic Search” button. The top score of this search was “evm.model.C153610.1” subject, indicating that the input sequence fragment has high similarity to the Salvia Miltiorrhiza protein. For more detailed use cases for this database, please refer to the Supplementary file.

In the future, we will collect more Chinese medicine genome data to provide data support for Chinese medicine research.

## Conclusions

Here, we reported a database of TCM-Blast database that integrates several database resources and markedly improves the efficiency of TCM genomic research. This database will allow users to perform batch sequence searches against integrated TCM genomic sequence databases. Therefore, TCM-Blast provided comprehensive Chinese medicine genome resource data on TCM scientific research and eliminates the latent redundancy occurring in other platforms.

## Supplementary Information

**Additional file 1: Figure S1**. Setting of protein sequence alignment options with Glycyrrhiza Uralensis protein database through the program of ‘blastp’. **Figure S2**. BLAST result of protein sequence alignment with Glycyrrhiza Uralensis protein database by inputting the query protein sequence. **Figure S3**. Setting of protein sequence alignment options with Glycyrrhiza Uralensis Nucleotide Database by the program of ‘tblastn’. **Figure S4**. BLAST result of protein sequence alignment with Glycyrrhiza Uralensis protein database by the program of ‘tblastn’. **Figure S5**. Setting of nucleotide sequence alignment options with Glycyrrhiza Uralensis Nucleotide Database through the program of ‘blastn’. **Figure S6**. BLAST result of nucleotide sequence alignment with Glycyrrhiza Uralensis nucleotide Database via the program of ‘blastn’. **Figure S7**. Setting of nucleotide sequence alignment options with Glycyrrhiza Uralensis Protein (Gancao) Database through the program of ‘blastx’. **Figure S8**. BLAST result of nucleotide sequence alignment with Glycyrrhiza Uralensis Protein (Gancao) Database via the program of ‘blastx’

## Data Availability

TCM-Blast is a free database and visualization tool open to all users with no login requirements and can be accessed at the following URL: http://viroblast.pungentdb.org.cn/TCM-Blast/viroblast.php. The web tool is functional on all modern web browsing environments including Google Chrome, Mozilla Firefox and Safari. All related species genomes data can be downloaded from http://viroblast.pungentdb.org.cn/TCM-Blast/db.

## Abbreviations

TCM: Traditional Chinese medicine
DNA: Deoxyribonucleic acid
TCM-Blast: Traditional Chinese medicine Basic Local Alignment Search Tool
