# Supplementary material for: TCM-Blast for traditional Chinese medicine genome alignment with integrated resources
Source: BMC Plant Biol. 2021 Jul 17;21:339. doi: 10.1186/s12870-021-03096-1 (PMC8285853; doi:10.1186/s12870-021-03096-1)

**TCM-Blast for traditional Chinese medicine genome alignment with integrated resources**

Zhao Chen^1,2^, Jing Li^1,2^, Ning Hou^1,2^ , Yanling Zhang^1,2^* and Yanjiang Qiao^1,2*^

^1^ School of Chinese Materia Medica, Beijing University of Chinese Medicine, Yangguang South Avenue, Fangshan District, Beijing, 102488, China

^2^ Research Center of TCM-Information Engineering, State Administration of Traditional Chinese Medicine of The Peoples Republic of China, Yangguang South Avenue, Fangshan District, Beijing, 102488, China

* To whom correspondence should be addressed. Tel: +86-010-8473-6150; Fax: +86-10-8473-8661; Email: [zhaochen@bucm.edu.cn](mailto:zhaochen@bucm.edu.cn)

**Example usage**

Users can then select Glycyrrhiza Uralensis’ protein database with the program of “blastp” to obtain their expected BLAST results via inputting the protein sequence. In **Fig. S1**, users input the protein sequence fragment:


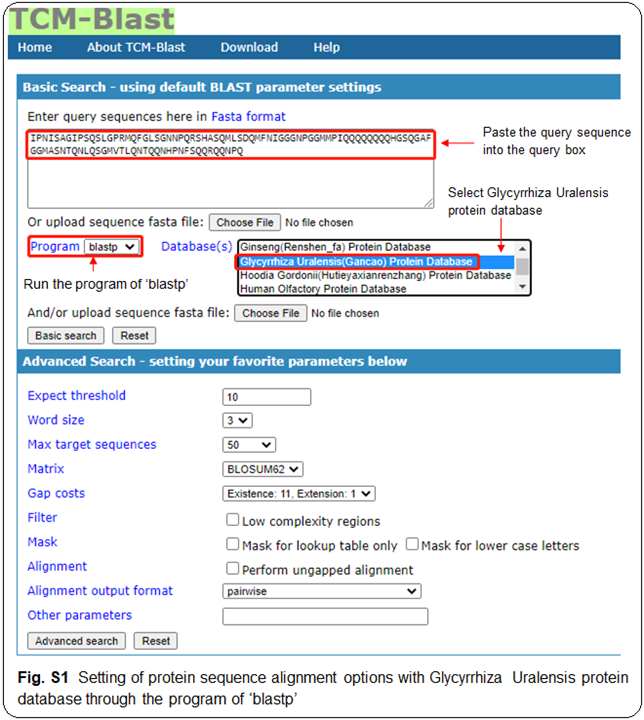


“*IPNISAGIPSQSLGPRMQFGLSGNNPQRSHASQMLSDQMFNIGGGNPGGMMPIQQQQQQQQHGSQGAFGGMASNTQNLQSGMVTLQNTQQNHPNFSQQRQQNPQ*” in “Enter query sequences” box; then select the Glycyrrhiza Uralensis protein database; the users can get the BLAST result by clicking ”Basic Search” button. The top score of this search is “Glyur008776s00045098.1” subject (**Fig. S2**), indicating the input sequence fragment is high similarity with the Glycyrrhiza Uralensis protein.


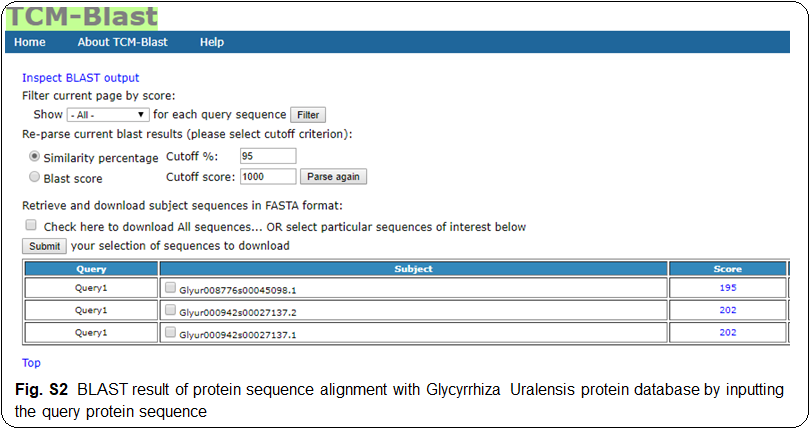


When the program of “tblastn” was selected with the same protein sequence in query box, the Glycyrrhiza Uralensis (Gancao_fa) Nucleotide Database would be selected (**Fig. S3**). Then, click the button of ”Basic search”. The program of “tblastn”can convert protein sequence into nucleic acid sequence, and then compare with Glycyrrhiza Uralensis Nucleotide Database. The results showed that the input protein sequence was consistent with scaffold 08776 and scaffold 00942 (**Fig. S4**).


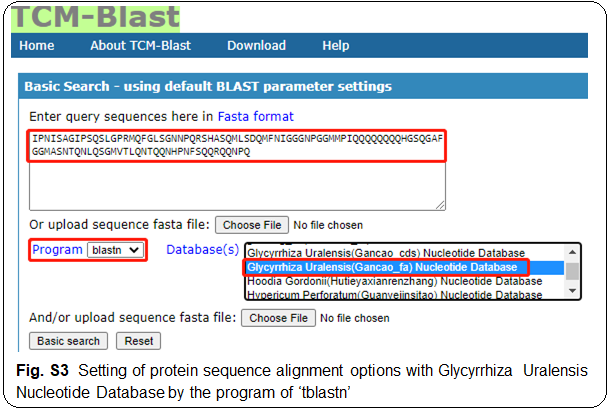


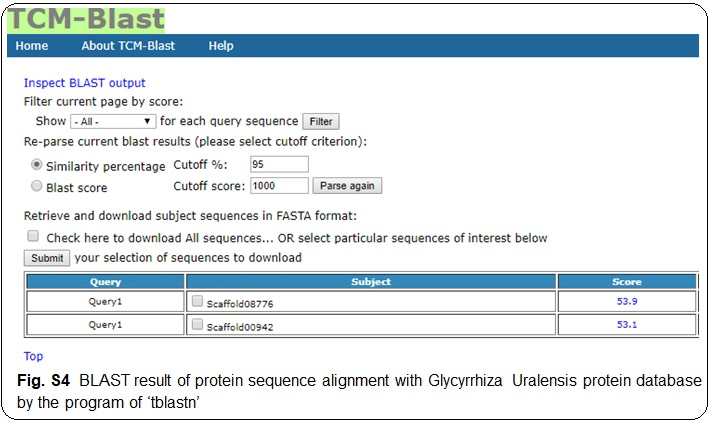


If users want to DNA sequence similarity searches by the means of inputting nucleotide sequence fragment, they can also select Glycyrrhiza Uralensis nucleotide database with the program of “blastn”


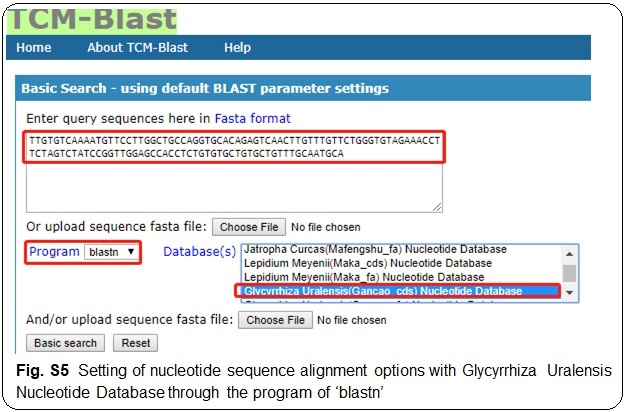


to obtain their expected BLAST results. In **Fig. S5**, users input the nucleotide sequence fragment:

“*TTGTGTCAAAATGTTCCTTGGCTGCCAGGTGCACAGAGTCAACTTGTTTGTTCTGGGTGTAGAAACCTTCTAGTCTATCCGGTTGGAGCCACCTCTGTGTGCTGTGCTGTTTGCAATGCA*” in “Enter query sequences” box; then select the Glycyrrhiza Uralensis Nucleotide Database; the users can get the BLAST result by clicking ”Basic search” button. The top score of this search is “Glyur000001s00000001.1” subject (**Fig. S6**), indicating the input sequence fragment is high similarity with the Glycyrrhiza Uralensis nucleotide.


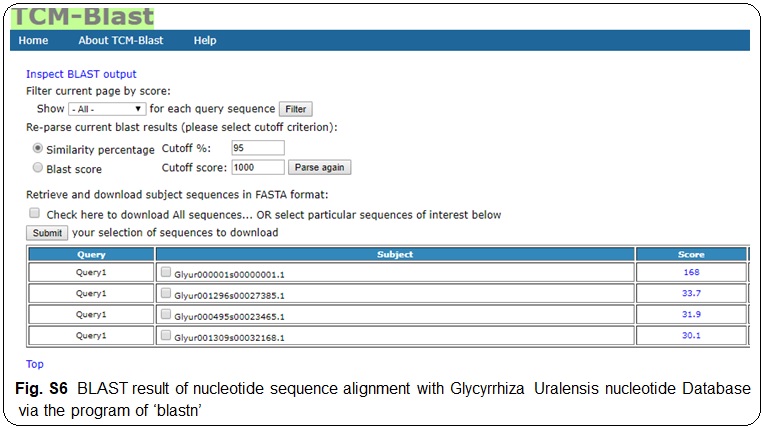


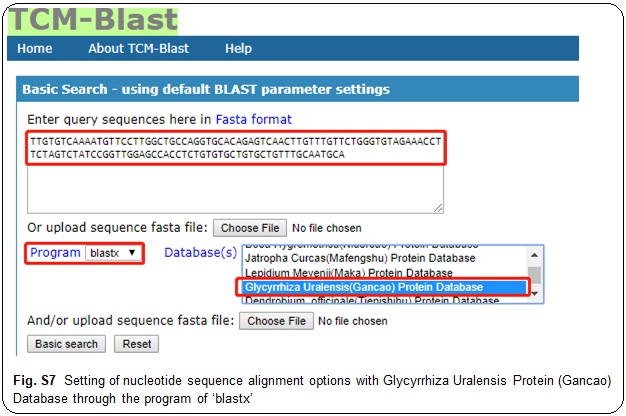


Users can obtain their expected sequence similarity search results through Glycyrrhiza Uralensis (Gancao) Protein Database with the program of “blastx”. In **Fig. S7**, users input the nucleotide sequence fragment:

“*TTGTGTCAAAATGTTCCTTGGCTGCCAGGTGCACAGAGTCAACTTGTTTGTTCTGGGTGTAGAAACCTTCTAGTCTATCCGGTTGGAGCCACCTCTGTGTGCTGTGCTGTTTGCAATGCA*” in “Enter query sequences” box; then select the Glycyrrhiza Uralensis (Gancao) Protein Database; the users can get the BLAST result by clicking ”Basic Search” button (**Fig. S7**). The top score of this search is “Glyur000001s00000001.1” subject (**Fig. S8**), indicating the input nucleotide sequence fragment is high similarity with the Glycyrrhiza Uralensis.


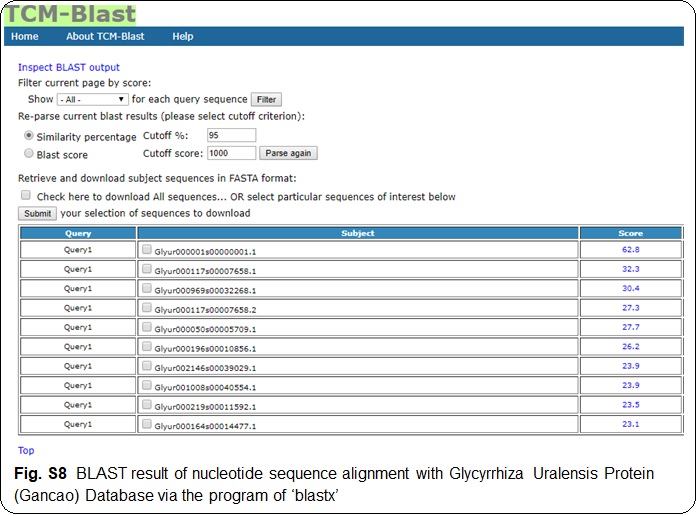

Supplement: Supplementary file 1 — Additional file 1: Figure S1. Setting of protein sequence alignment options with Glycyrrhiza Uralensis protein database through the program of ‘blastp’. Figure S2. BLAST result of protein sequence alignment with Glycyrrhiza Uralensis protein database by inputting the query protein sequence. Figure S3. Setting of protein sequence alignment options with Glycyrrhiza Uralensis Nucleotide Database by the program of ‘tblastn’. Figure S4. BLAST result of protein sequence alignment with Glycyrrhiza Uralensis protein database by the program of ‘tblastn’. Figure S5. Setting of nucleotide sequence alignment options with Glycyrrhiza Uralensis Nucleotide Database through the program of ‘blastn’. Figure S6. BLAST result of nucleotide sequence alignment with Glycyrrhiza Uralensis nucleotide Database via the program of ‘blastn’. Figure S7. Setting of nucleotide sequence alignment options with Glycyrrhiza Uralensis Protein (Gancao) Database through the program of ‘blastx’. Figure S8. BLAST result of nucleotide sequence alignment with Glycyrrhiza Uralensis Protein (Gancao) Database via the program of ‘blastx’ [file 12870_2021_3096_MOESM1_ESM.doc]
